# Sorbate Transport in Carbon Molecular Sieve Membranes and FAU/EMT Intergrowth by Diffusion NMR

**DOI:** 10.3390/ma5020302

**Published:** 2012-02-14

**Authors:** Robert Mueller, Rohit Kanungo, Amrish Menjoge, Mayumi Kiyono-Shimobe, William J. Koros, Steven A. Bradley, Douglas B. Galloway, John J. Low, Sesh Prabhakar, Sergey Vasenkov

**Affiliations:** 1 Department of Chemical Engineering, University of Florida, Gainesville, FL 32611, USA; E-Mails: silveram@ufl.edu (R.M.); rohit.kanungo@ufl.edu (R.K.); amrishm@ufl.edu (A.M.);; 2 School of Chemical & Biomolecular Engineering, Georgia Institute of Technology, Atlanta, GA 30332, USA; E-Mails: mayumi.marbury@gmail.com (M.K.-S.); bill.koros@chbe.gatech.edu (W.J.K.); 3 UOP LLC, A Honeywell Company, 25 E. Algonquin Road, Des Plaines, IL 60017-5017, USA; E-Mails: steven.bradley@uop.com (S.A.B.); douglas.galloway@uop.com (D.B.G.); jlow@mcs.anl.gov (J.J.L.); sesh.prabhakar@uop.com (S.P.)

**Keywords:** carbon molecular sieve, zeolite, FAU/EMT intergrowth, diffusion, NMR

## Abstract

In this paper we present and discuss selected results of our recent studies of sorbate self-diffusion in microporous materials. The main focus is given to transport properties of carbon molecular sieve (CMS) membranes as well as of the intergrowth of FAU-type and EMT-type zeolites. CMS membranes show promise for applications in separations of mixtures of small gas molecules, while FAU/EMT intergrowth can be used as an active and selective cracking catalyst. For both types of applications diffusion of guest molecules in the micropore networks of these materials is expected to play an important role. Diffusion studies were performed by a pulsed field gradient (PFG) NMR technique that combines advantages of high field (17.6 T) NMR and high magnetic field gradients (up to 30 T/m). This technique has been recently introduced at the University of Florida in collaboration with the National Magnet Lab. In addition to a more conventional proton PFG NMR, also carbon-13 PFG NMR was used.

## 1. Introduction

Detailed understanding of diffusion of guest molecules in microporous materials is important for numerous industrial applications, which include separations, catalysis, and detection of chemically or biologically important species. In this work we review results of our recent studies of sorbate self-diffusion in two types of microporous materials, *viz.* carbon molecular sieve (CMS) membranes and the intergrowth of FAU-type and EMT-type zeolites.

CMS membranes are amorphous microporous carbon materials formed by pyrolysis of polymer precursors. The pore system of CMS membranes consists of selective ultra-micropores (<6 Å) and slightly larger micropores (6–20 Å) [1,2]. Carbon molecular sieve membranes represent a promising class of materials for energy efficient separations of gas mixtures consisting of similarly sized molecules such as N_2_/O_2_ and CO_2_/CH_4_ due to the diffusion dominant process [3,4]. Selectivities of CMS membranes for such difficult separations approach those of zeolite and metal organic framework (MOF) membranes. At the same time, costs of large-scale fabrication of defect-free CMS membranes are expected to be much lower than those of zeolite and MOF membranes. CMS membranes are also less brittle than most zeolite and MOF membranes.

Previous experimental investigations of sorbate transport in CMS membranes have been performed using macroscopic techniques, which for the most part are based on membrane permeability measurements [2,5,6,7]. To our knowledge, until now microscopic studies of diffusion of light gases in a broad range of length scales inside CMS membranes were not reported. To fill this gap, we review our first data on sorbate self-diffusion on the micrometer and sub-micrometer length scales inside CMS membranes.

**Figure 1:**
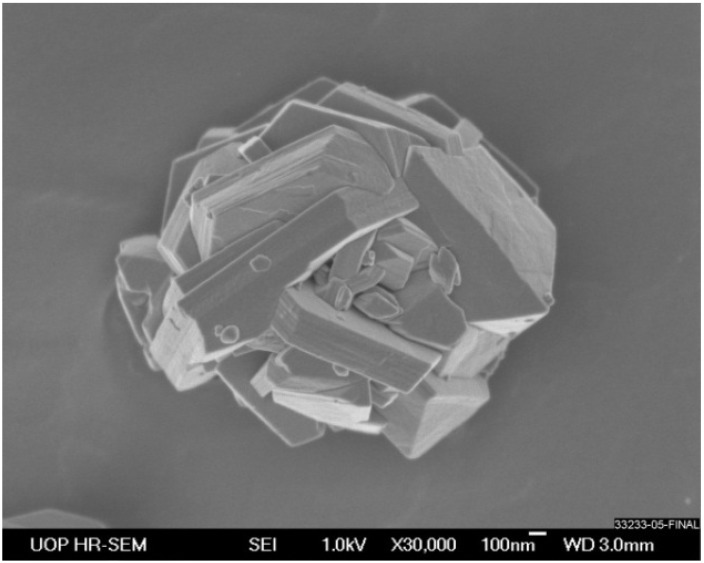
SEM image of a representative particle of FAU/EMT intergrowth.

The other focus of this work is to review our recent results on sorbate self-diffusion in the intergrowth of FAU and EMT-type zeolites. Molecular transport in FAU-type zeolites has been extensively investigated using a number of experimental techniques. As a result, detailed data on intracrystalline diffusivities of different types of sorbate molecules are available for this zeolite [8,9]. At the same time, much less is known about diffusion properties of FAU/EMT intergrowth. The origin of the structural differences between FAU and EMT zeolites is related to the type of stacking of sodalite layers. In the FAU-type zeolite the stacking occurs in a ABCABC sequence, while in the EMT-type zeolite it occurs in a ABABAB sequence [10]. Figure 1 shows SEM image of a typical particle of FAU/EMT intergrowth used for diffusion studies. This particle can be described as a sandwich of several EMT-type blocks, which resemble thin hexagonal plates, and FAU-type blocks, which have a typical for this zeolite octahedral shape [11,12]. An overall diffusion of sorbate molecules in such sandwich-like particles can be slowed down by reduced transport rates through the interfaces between FAU and EMT blocks. References [13,14,15,16,17] provide examples of the observation of a similar decrease of the effective diffusivities inside zeolite crystals due to the existence of intracrystalline defects and the resulting transport barriers.

Microscopic diffusion studies in CMS membranes and FAU/EMT intergrowth were carried out by high field and high gradient PFG NMR. The diffusion measurements were performed at different temperatures and for different diffusion times corresponding to different mean square displacements (MSDs) of sorbate molecules. The results reported below demonstrate that these measurements show high potential for uncovering the relationship between structural properties and transport in materials with a complex micropore structure.

## 2. Details of PFG NMR Measurements and Data Processing

Diffusion studies were performed by proton and carbon-13 PFG NMR using a wide-bore 17.6 T Bruker BioSpin spectrometer. Magnetic field gradients with the amplitude up to 30 T/m were generated using Diff60 diffusion probe and Great60 gradient amplifier (Bruker BioSpin). The use of high field affords high sensitivity and the ability to apply high gradients allows the possibility to measure self-diffusion coefficients of sorbate molecules in micropores on small (*i.e.*, sub-micrometer) length scales of molecular displacements.

Diffusion measurements were carried out using the 13-interval PFG NMR sequence with bipolar gradients [18] and the PFG NMR stimulated echo sequence with the longitudinal eddy current delay (PGSTE LED) [19]. The former sequence allows reducing or even completely eliminating the disturbing influence of magnetic susceptibility effects. Susceptibility effects are expected for heterogeneous porous materials such as stacks of membrane pieces and zeolite beds studied in this work. The advantage of the latter sequence is related to the possibility of reducing influence of the transverse (*T*_2_) NMR relaxation on the measured signal. Such reduction is achieved by keeping short the sequence time intervals during which this relaxation process reduces the measured signal. For PGSTE LED sequence the absence of disturbing susceptibility effects and other measurement artifacts was confirmed by using the following two strategies: (i) it was verified that proton and carbon-13 PFG NMR diffusion data measured for the same diffusing species in the same samples and under the same conditions coincide within the experimental uncertainty, and (ii) it was checked that an increase of the time interval between the first and second π/2 radiofrequency pulses of PGSTE LED sequence (while keeping the effective diffusion time constant) does not change the measured diffusivities.

^13^C PFG NMR was employed in addition to the more traditional ^1^H PFG NMR to take advantage of the longer ^13^C longitudinal (*T_1_*) and transverse (*T_2_*) relaxation times than those typically observed for protons of guest molecules confined in nanopores. An increase in the former relaxation time allows measurements in a broader range of diffusion times and hence broader range of MSDs, while an increase in the later significantly improves the strength of the measured PFG NMR signal. Due to the small gyromagnetic ratio (*γ*) of carbon-13 (about ¼ of proton), the advantages of using high field and high gradients are especially important because a reduction in *γ* is expected to lead to reductions in the signal-to-noise ratio of an NMR signal as well as in the smallest length scale of molecular displacements for which PFG NMR diffusion measurements are still possible.

The 13-interval and PGSTE LED sequences were used to measure PFG NMR attenuation curves, *i.e.*, dependencies of the intensity of PFG NMR signal (*A*) on the amplitude of the magnetic field gradient (*g*). The signal intensity was obtained by measuring amplitude of NMR lines of the sorbate molecules or area under these lines. The proton and carbon-13 NMR spectra of each type of sorbate consisted of a single line. It was verified that within the experimental uncertainty the line shape does not depend on the gradient amplitude. The PFG NMR attenuation curves provide direct information on effective diffusivities. Effective diffusivities *D_i_* were obtained from monoexponential or biexponential fit of the PFG NMR attenuation curves using:

(1) Ψ≡A(g)A(g=0)=∑i=1n=1or2pi exp(−16q2〈ri2(t)〉)=∑i=1n=1or2pi exp(−q2tDi)

where *q* = *γδg* for PGSTE LED sequence and *q* = 2*γδg* for the 13-interval sequence, *γ* is the gyromagnetic ratio, *δ* denotes the duration of the field gradient pulses, and *t_eff_* is the effective diffusion time which corresponds to the diffusion time (or observation time) in a PFG NMR experiment. $\langle r_{i}^{2}(t)\rangle$ and *p_i_* denote, respectively, the mean square displacement (MSD) and fraction of sorbate molecules diffusing with a diffusivity *D_i_*. Monoexponential behavior (*n* = 1 in Equation (1)) corresponds to the situation when all molecules under study diffuse with a single effective diffusivity. In the case of biexponential behavior (*n* = 2 in Equation (1)) there are two ensembles of molecules diffusing with two different diffusivities. The expression in the right-hand part of Equation (1) was obtained using the Einstein relation:

(2) $$\langle r_{i}^{2}(t)\rangle =6D_{i}t$$

values of *δ* and *t_eff_* were kept constant for measurement of each attenuation curve.

## 3. Results and Discussion

### 3.1. Methane Self-Diffusion in CMS Membranes

**Figure 2:**
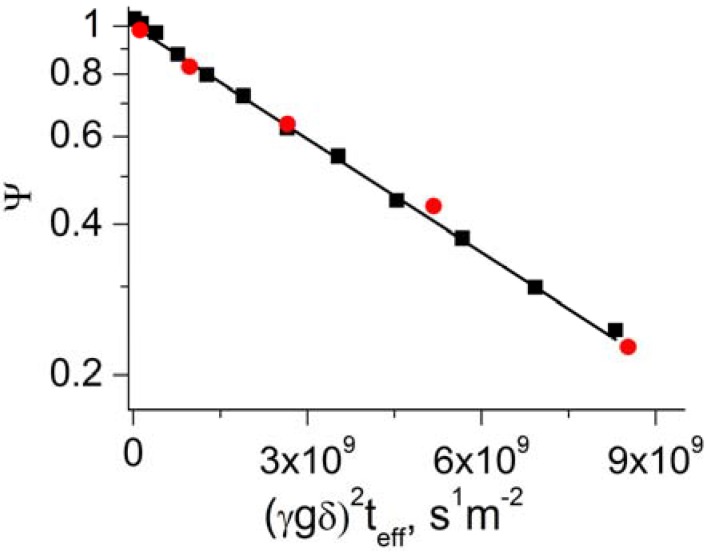
Examples of PFG NMR attenuation curves measured for methane diffusion in 6FDA/BPDA using proton (■) and carbon-13 (●) PFG NMR. The measurements were performed for the effective diffusion time of 9 ms at T = 321 K. The 13-interval and PGSTE LED PFG NMR pulse sequences were used for proton and carbon-13 measurements, respectively. The line shows the result of fitting both attenuation curves by Equation (1) with n = 1.

Methane self-diffusion was investigated by PFG NMR in the following three carbon molecular sieve membrane samples: 6FDA/BPDA(1:1)-DAM CMS pyrolized in an inert gas (6FDA/BPDA), Matrimid^®^ CMS pyrolized in an inert gas (Matrimid Sample 1), and Matrimid^®^ CMS pyrolized under vacuum (Matrimid Sample 2). Diffusion data was obtained using combined application of proton and carbon-13 PFG NMR. It was observed that in all cases the PFG NMR attenuation curves show monoexponential behavior in agreement with Equation (1), *n* = 1 (Figure 2). This indicates that under any given measurement conditions used there is no significant distribution over methane self-diffusion coefficient inside membrane pieces of each studied membrane sample. It is important to note that under our experimental conditions the diffusivity of methane in the gas phase of the samples is expected to be at least four orders of magnitude larger than that inside the membranes. This estimate was confirmed by our direct PFG NMR measurements. As a result, the PFG NMR signal from methane in the gas phase was completely attenuated when the smallest (non-zero) gradient was used in the measurements of the PFG NMR attenuation curves shown in Figure 2. It was also observed that the diffusion data for methane measured under the same conditions by ^1^H and ^13^C PFG NMR were identical within the experimental uncertainty. Figure 2 shows an example of such data. In the presentation of this figure the proton and carbon-13 PFG NMR attenuation curves measured for diffusion of ^13^C-labeled CH_4_ have to coincide to reveal the same diffusion behavior. Such coincidence is evident in the figure. The observed agreement between the diffusivities measured at very different resonance frequencies of 750 and 188.6 MHz corresponding, respectively, to ^1^H and ^13^C nuclei provide strong evidence that the reported diffusion data are not distorted by magnetic susceptibility effects or by any other disturbing effects.

**Figure 3:**
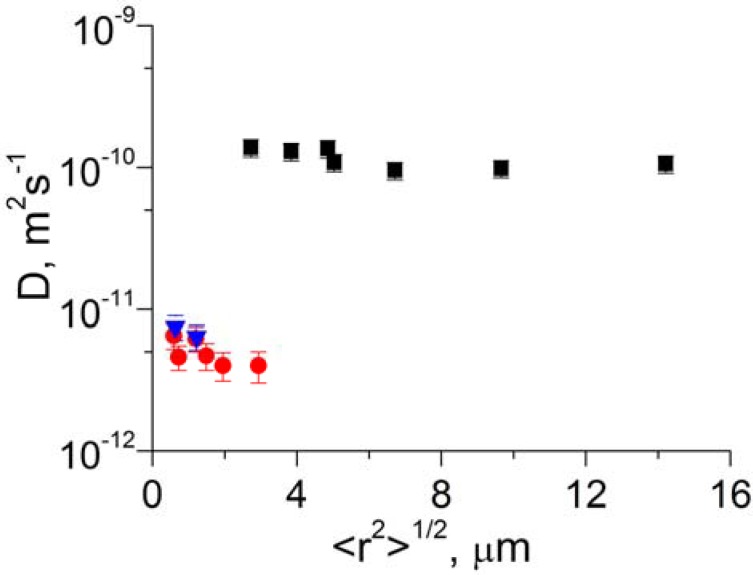
Dependences of the methane self-diffusion coefficient on the root MSD at *T* = 297 K for 6FDA/BPDA (■), Matrimid Sample 1 (●), and Matrimid Sample 2 (▼). The data was obtained using Equation (1) with *n* = 1 and Equation (2).

Table 1 shows the methane diffusivities and the corresponding values of root MSD obtained by fitting the measured PFG NMR attenuation curves by Equation (1) (*n* = 1) and Equation (2). The diffusion data are reported for the broad range of temperatures and effective diffusion times used in this study. In all cases the reported values of root MSD were at least 5 times smaller than the smallest dimension (*i.e.*, thickness) of the membrane pieces. Hence, the measured diffusivities correspond to intra-membrane diffusion under conditions when the boundaries of the membrane pieces are not expected to influence the diffusion behavior. In this case it is not expected to observe any measurable fraction of methane molecules performing long-range diffusion, *i.e.*, diffusion under conditions of fast exchange of methane molecules between the membrane pieces and the surrounding gas phase. It is seen in Table 1 that within the experimental uncertainty the intra-membrane diffusivities of methane do not change with increasing values of the root MSD. This is illustrated in Figure 3 which presents examples of the measured dependencies of the methane diffusivities on the root MSD. This data demonstrates that the diffusion properties of the studied membranes remain homogeneous over a broad range of length scales starting with the length scales as small as 600 nm to as large as 14 µm which is comparable with the membrane thickness of 60 ± 10 µm.

Table 1Data for methane self-diffusion in: (**a**) 6FDA/BPDA, and (**b**) Matrimid Samples 1 and 2. Self-diffusion coefficients and the corresponding values of the root MSD were obtained from fitting PFG NMR attenuation curves measured for methane diffusion at different temperatures (*T*) and effective diffusion times (*t_eff_*) by Equation (1) with *n* = 1 and Equation (2). The data obtained by ^13^C PFG NMR is labeled by (*). All other data was obtained by ^1^H PFG NMR.

**Table:** (**a**)

Sample	*T* (K)	*t_eff_* (ms)	*D* (m^2^·s^−1^)	Root MSD (µm)
6FDA/BPDA loaded with methane	297	9	(1.4 ± 0.2) × 10^−10^	2.7 ± 0.2
		9	(1.2 ± 0.2) × 10^−10 ^ *	2.6 ± 0.2
		19	(1.3 ± 0.2) × 10^−10^	3.8 ± 0.3
		19	(1.3 ± 0.2) × 10^−10^ *	3.8 ± 0.3
		29	(1.4 ± 0.2) × 10^−10^ *	4.9 ± 0.3
		39	(1.2 ± 0.2) × 10^−10^	5.3 ± 0.4
		39	(1.2 ± 0.2) × 10^−10^ *	5.3 ± 0.4
		79	(9.5 ± 1.3) × 10^−11^ *	6.7 ± 0.5
		159	(9.8 ± 1.4) × 10^−11^ *	9.7 ± 0.7
		319	(1.1 ± 0.1) × 10^−10^ *	14.0 ± 1.0
	319	9	(1.7 ± 0.2) × 10^−10^	3.0 ± 0.2
		9	(1.7 ± 0.2) × 10^−10^ *	3.0 ± 0.2
		19	(1.5 ± 0.2) × 10^−10^	4.1 ± 0.3
		39	(1.4 ± 0.2) × 10^−10^	5.7 ± 0.4
		39	(1.5 ± 0.2) × 10^−10^ *	5.9 ± 0.4
	340	9	(2.2 ± 0.3) × 10^−10^	3.4 ± 0.2
		9	(2.1 ± 0.3) × 10^−10^	3.4 ± 0.2
		19	(2.2 ± 0.3) × 10^−10^	5.0 ± 0.3
		39	(2 ± 0.3) × 10^−10^ *	6.8 ± 0.5
	353	9	(2.7 ± 0.4) × 10^−10^	3.8 ± 0.3
		9	(2.8 ± 0.4) × 10^−10^ *	3.9 ± 0.3
		19	(2.6 ± 0.4) × 10^−10^	5.4 ± 0.4
		39	(2.4 ± 0.3) × 10^−10^	7.5 ± 0.5

**Table:** (**b**)

Sample	*T* (K)	*t_eff_* (ms)	*D* (m^2^·s^−1^)	Root MSD (µm)
Matrimid Sample 1 loaded with methane	297	9	(6.5 ± 1.3) × 10^−12^	0.6 ± 0.06
		19	(4.6 ± 0.9) × 10^−12^	0.7 ± 0.1
		39	(6.2 ± 1.2) × 10^−12^	1.2 ± 0.1
		79	(4.7 ± 1) × 10^−12^	1.5 ± 0.2
		159	(4 ± 0.9) × 10^−12^	2.0 ± 0.2
		359	(4 ± 1) × 10^−12^	2.9 ± 0.4
	339	9	(9.4 ± 2) × 10^−12^	0.7 ± 0.07
	356	9	(1.2 ± 0.2) × 10^−11^	0.8 ± 0.05
Matrimid Sample 2 loaded with methane	297	9	(7.5 ± 1.5) × 10^−12^	0.6 ± 0.1
		39	(6.4 ± 1.3) × 10^−12^	1.2 ± 0.1
	336	9	(8.5 ± 1.5) × 10^−12^	0.7 ± 0.1
	356	9	(1.3 ± 0.2) × 10^−11^	0.8 ± 0.1

**Figure 4:**
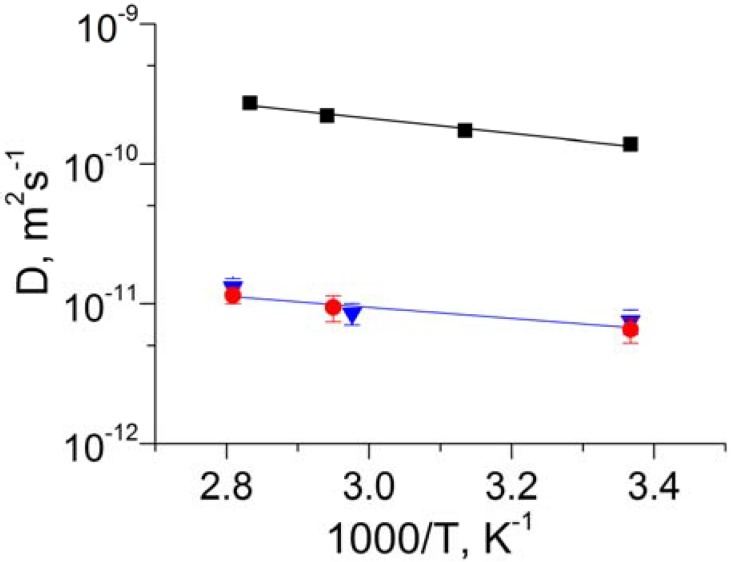
Temperature dependences of the methane self-diffusion coefficient measured by PFG NMR for the effective diffusion time 9 ms in 6FDA/BPDA (■), Matrimid Sample 1 (●), and Matrimid Sample 2 (▼). Lines show the fit to the Arrehenius law (Equation (3)).

The temperature dependence of the methane self-diffusivities in the three membrane samples is shown in Figure 4. For each sample, the temperature dependence was found to be in agreement with an Arrhenius law

(3) $$D(T)=D_{0}\;\text{exp}(-E_{a}/RT)$$

where *E_a_* is the apparent activation energy of diffusion. Application of Equation (3) to the measured data yields the following activation energies: 8 kJ/mol for 6FDA/BPDA and 10 kJ/mol for Matrimid Sample 1 and Matrimid Sample 2. The experimental uncertainty of these values was estimated to be in the range of a factor of 2.

It is seen in Table 1 and Figure 4 that under the same or similar measurement conditions the methane self-diffusivity in 6FDA/BPDA is about an order of magnitude larger than that in both Matrimid samples. At the same time, in all three samples the activation energy for methane diffusion was found to be the same within the experimental uncertainty. This observation can be explained by assuming that 6FDA/BPDA has a higher relative abundance of sufficiently large pores permeable to methane than the Matrimid samples. This is in agreement with the data on permeation experiments conducted on these membrane types and with the data analysis reported in references [5,20]. In a qualitative agreement with the self-diffusion data reported in Table 1 the permeation experiments yielded the effective transport diffusivities of methane that are much lower in Matrimid^®^ type CMS than in 6FDA/BPDA type CMS [5]. The quantitative comparison of the transport diffusivities with the self-diffusivities reported in this work cannot be done easily because the transport diffusivities represent the effective (average) values over a broad range of methane loadings in the membranes corresponding to a large methane pressure difference (around 50 psia) between the feed side and the permeate side of the membranes. Hence, both the thermodynamic factor and loading dependence of diffusivity are expected to contribute to the difference between the transport and self-diffusivity values [8,9].

### 3.2. Self-Diffusion of Isooctane in FAU/EMT Intergrowth

**Figure 5:**
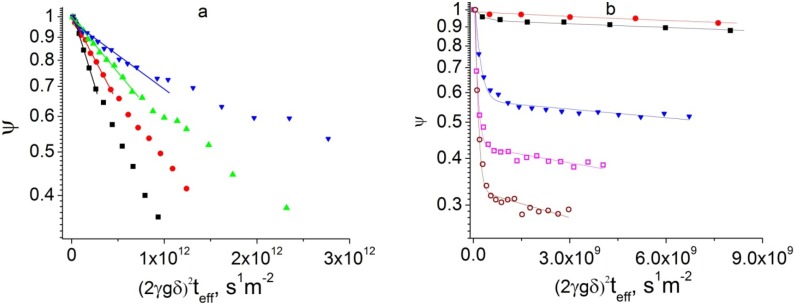
(**a**) Proton PFG NMR attenuation curves measured by the 13-interval PFG NMR sequence for diffusion of isooctane in the FAU/EMT intergrowth at *T* = 264 K for the following effective diffusion times: 6.0 ms (■), 10.5 ms (●), 20.5 ms (▲), and 40.5 ms (▼). The lines show the initial slopes of the attenuation curves; (**b**) Proton PFG NMR attenuation curves measured by the 13-interval PFG NMR sequence for diffusion of isooctane in the FAU/EMT intergrowth at *T* = 289 K for the following effective diffusion times: 5.7 ms (■), 10.2 ms (●), 40.1 ms (▼), 80.1 ms (□), and 120.1 ms (○). The lines show the best fit curves of the measured data by Equation (1) with *n* = 2.

Figure 5a shows examples of the PFG NMR attenuation curves measured for isooctane diffusion in the FAU/EMT intergrowth for different effective diffusion times at 264 K. In the presentation of this figure the initial parts of the attenuation curves measured for different effective diffusion times have to coincide if the effective diffusivity, which can be defined by the initial slope of the attenuation curves [8], does not depend on diffusion time. The data in Figure 5a show that the initial slope of the attenuation curves decreases with increasing effective diffusion time. This indicates that the effective diffusivity of isooctane decreases as the diffusion time increases. The shape of the attenuation curves in the figure is relatively close to mono-exponential in agreement with Equation (1), *n* = 1. The results of fitting of the initial parts of the attenuation curves in Figure 5a by this equation are shown in the figure and also given in Table 2. It is important to note that while the effective diffusivity continuously decreases with increasing diffusion time, the values of the root MSD remain independent of diffusion time within the experimental uncertainty (Table 2). The latter values are much smaller than the characteristic size of the particles of the FAU/EMT intergrowth (around 2.8 microns). At the same time, the root MSD values are comparable with the sizes of the individual FAU and EMT blocks forming these particles. Figure 1 shows that the block sizes are between around 0.1 and 1 micron.

**Table 2:** Results of fitting of the initial parts of the attenuation curves in Figure 5a by Equation (1), *n* = 1 and the corresponding effective diameters of the particle components surrounded by transport barriers *d_eff_*. The values of *d_eff_* were obtained by using Equation (4).

Sample	*T* (K)	*t*_eff_ (ms)	*D_eff_* (m^2^·s^−1^)	Root MSD (µm)	*d_eff_* (µm)
FAU/EMT intergrowth loaded with isooctane	264	6.0	(1.6 ± 0.2) × 10^−12^	0.24 ± 0.2	0.44 ± 0.4
		10.5	(9.0 ± 1.4) × 10^−13^	0.24 ± 0.2	0.44 ± 0.4
		20.5	(5.7 ± 0.9) × 10^−13^	0.26 ± 0.2	0.48 ± 0.5
		40.5	(2.7 ± 0.4) × 10^−13^	0.26 ± 0.2	0.48 ± 0.5

These results can be explained by assuming transport barriers at the interfaces between intergrowth components in the particles of the FAU/EMT intergrowth. These barriers are essentially impermeable for isooctane molecules for diffusion times up to 41 ms at 264 K, as indicated by the unchanging values of the root MSD in Table 2. An existence of a distribution over the sizes of the particle components surrounded by the transport barriers explains small deviations of the PFG NMR attenuation curves in Figure 5a from the monoexponential behavior [8]. Assuming that all particle components separated by the transport barriers can be approximated by spheres with the diameter *d_eff_*, the following expression can be used to estimate the value of *d_eff_* [8,21,22].

(4) $$D_{eff}=\frac{\langle r{\left(t\right)}^{2}\rangle }{6t}=\frac{D_{eff}^{2}}{20t}$$

Table 2 presents the values of *d_eff_* that were obtained using Equation (4). It is seen that these values obtained for different diffusion times are all the same within the experimental uncertainty. The values of *d_eff_* characterize the mean size of the particle components surrounded by the transport barriers. This size is found to be within the range of the observed sizes of the intergrowth components (Figure 1). Hence, these data indicate that the intergrowth components are surrounded by the observed transport barriers.

Figure 5b shows examples of the measured PFG NMR attenuation curves for isooctane in the FAU/EMT intergrowth at a higher temperature of 289 K. It is seen in Figure 5b that the attenuation curves at large diffusion times show fast initial decay, which is followed by much slower signal attenuation at larger gradient strengths. The onset of the slow attenuation shifts to higher attenuation values with increasing diffusion time. Such behavior indicates a transition of the diffusion process from the intraparticle to long-range regimes as diffusion time increases [8]. In the former regime sorbate displacements are smaller than or comparable with the particle sizes, while in the latter regime these displacements are larger than the particle sizes. As a result, with increasing diffusion time the fraction of molecules corresponding to intraparticle diffusion is expected to decrease, while that corresponding to long-range diffusion is expected to increase. The sorbate fractions and diffusivities corresponding to intraparticle (slow attenuation) and long-range (fast attenuation) diffusion can be obtained from the biexponential fit of the attenuation curves (Equation (1) with *n* = 2). Table 3 presents the resulting diffusivities and molecular fractions for intraparticle diffusion (*D*_2_ and *p_2_*) and for long-range diffusion (*D*_1_ and *p_1_*).

**Table 3:** Results of fitting of the PFG NMR attenuation curves measured for isooctane diffusion in the FAU/EMT intergrowth at *T* = 289 K by Equation (2) with *n* = 2. The intraparticle diffusivities (*D_2_*) were obtained from the attenuation curves measured at the extended gradient range (not shown in Figure 5). Star (*) indicates values with a large (in the range of a factor of 2–4) experimental uncertainty.

Sample	*T*(K)	*t_eff_* (ms)	*D_1_* (m^2^·s^−1^)	*p_1_*	Root MSD 1 (µm)	*D_2_* (m^2^·s^−1^)	*P_2_*	Root MSD 2 (µm)
FAU/EMT intergrowth loaded with isooctane	289	5.7	4 × 10^−9^ *	0.1 ± 0.07	11 *	(3.5 ± 0.5) × 10^−12^	0.9 ± 0.07	0.34 ± 0.02
		10.2	2 × 10^−9^ *	0.1 ± 0.07	11 *	(2.5 ± 0.4) × 10^−12^	0.9 ± 0.07	0.39 ± 0.03
		40.1	(6 ± 3) × 10^−9^	0.5 ± 0.1	39 ± 10	(1.5 ± 0.2) × 10^−12^	0.5 ± 0.1	0.43 ± 0.03
		80.1	(1.1 ± 0.3) × 10^−8^	0.7 ± 0.1	74 ± 11	(4.0 ± 0.6) × 10^−13^	0.3 ± 0.1	0.44 ± 0.04
		120.1	(1.3 ± 0.5) × 10^−8^	0.8 ± 0.1	96 ± 20	(3.5 ± 0.5) × 10^−13^	0.2 ± 0.1	0.34 ± 0.02

It is seen in the table that within the experimental uncertainty the values of long-range diffusivities (*D*_1_) do not depend on diffusion time. At the same time, the fraction of molecules (*p*_1_) corresponding to long-range diffusion increases significantly with increasing diffusion time. This is an expected behavior for the transition from the intraparticle to long-range diffusion regime with increasing diffusion time [8]. Also, in agreement with the assignment of the ensemble 1 to molecules exhibiting long-range diffusion, the values of the root MSD for this ensemble (Table 3) are larger than the characteristic size of the particles of the FAU/EMT intergrowth (~2.8 μm). For diffusion in zeolite beds the long-range diffusivity often can be estimated (in the first approximation) as the diffusivity in the gas phase of the bed multiplied by the fraction of diffusing molecules located in the gas phase of the bed at any particular point of time. As a result, the values of long-range diffusivities can be several orders of magnitude larger than those for diffusion inside zeolite particles [8]. It is seen in Table 3 that the effective intraparticle diffusivities continuously decrease with increasing diffusion time. This shows that also at a higher temperature of 289 K the intraparticle diffusion of isooctane has a mostly restricted character. The values of the root MSD obtained for this diffusion process increase slowly with increasing diffusion time. Hence, in contrast to the behavior observed at 264 K, under our measurement conditions at 289 K isooctane molecules show an ability to gradually penetrate through the intraparticle transport barriers. The latter is also manifested by the existence of the molecular fraction exhibiting long-range diffusion. The dependence of this fraction on diffusion time shown in Table 3 allows estimating the permeability of the transport barriers that were most clearly observed experimentally at 264 K. This permeability can be obtained in the framework of the tracer desorption technique [8] by estimating the value of the intraparticle mean life time (*τ_intra_*):

(5) $$\tau_{intra}=\int_{0}^{\infty }\left(1-p_{1}(t_{eff})\right)dt_{eff},$$

where, in addition to the values of *p_1_* given in Table 3, one can use that *p_1_*(0) = 0 and *p_1_*(∞) = 1. The value of *τ_intra_* can be estimated using Equation (5) and taking into account that for the tracer exchange processes strongly influenced by the penetration through the transport barriers [8]. Using this expression it was found that *τ_intra_* = 104 ± 10 ms at 289 K. The intraparticle mean life time is related to the corresponding permeability (*k_p_*) of the transport barriers p1(teff)≈1−exp(−teffτintra) [8], which separate the particle components with the characteristic diameters equal to *d_eff_*

(6) $$\tau_{intra}\geq \frac{d_{eff}}{6k_{p}}$$

This relation is justified by the expectation that on their way out of the zeolite particles isooctane molecules have to cross over at least one of the transport barriers separating the particle components with the characteristic diameter *d_eff_*. Using Equation (6) we find *k_p_* ≥ 0.8 × 10^−6^ m/s at 289 K.

It is of interest to compare intraparticle diffusivities of isooctane in the FAU/EMT intergrowth with the corresponding diffusivities in the pure FAU and pure EMT zeolites. Clearly, in the particles of the pure zeolites there is no intergrowth of different zeolite types and no corresponding transport barriers at the interfaces between alternating FAU and EMT blocks. PFG NMR diffusion measurements of isooctane diffusion in the pure zeolites were performed using the 13-interval PFG NMR. The results of these measurements are shown in Table 4. These values were obtained in the same way as discussed above by fitting the measured PFG NMR curves using Equation (1) with *n* = 2 for a transition range between the intraparticle and long-range diffusion. The data for the pure zeolites were obtained at the same or comparable temperatures as those used in the studies of the FAU/EMT intergrowth. In all cases no dependence of the intraparticle diffusivities on diffusion time was observed for the pure zeolite samples. The lack of such dependence indicates that for the measured range of the root MSDs there are no significant diffusion restrictions by transport barriers inside the particles of the pure zeolite samples. The isooctane diffusivity in the FAU-type zeolite was found to be in a good agreement with the previously reported data [23].The effective intraparticle diffusivities of isooctane in the FAU and EMT samples were found to be significantly higher than that in the FAU/EMT intergrowth for large diffusion times at 264 K (compare data in Table 2 and Table 4). Clearly, this is in agreement with the conclusion that the isooctane diffusion in the FAU/EMT intergrowth at 264 K is strongly influenced by the intraparticle transport resistances resulting in lower effective intraparticle diffusivities in the intergrowth in comparison to those in the pure zeolites.

**Table 4:** Intracrystalline diffusivities of isooctane in the FAU and EMT zeolites. The diffusivities were obtained for the shown range of the effective diffusion times and the corresponding values of the root MSD by fitting the measured PFG NMR attenuation curves using Equation (1) with *n* = 2.

Sample	*T* (K)	*D* (m^2^·s^−1^)	*t_eff_* (ms)	Root MSD (µm)
FAU loaded with isooctane	266	(3.7 ± 0.7) × 10^−12^	9.5–39.5	0.5–0.9
EMT loaded with isooctane	264	(1.7 ± 0.3) × 10^−12^	9.2–39.2	0.3–0.6

It is quite likely that the intraparticle transport barriers, which are reported in this paper, are located at the interfaces between intergrowth components of the FAU/EMT intergrowth. Similar intracrystalline transport barriers were observed previously in other zeolite types [13,14,15,16]. It can be assumed that the reason for the existence of the barriers is a partial blockage of the channel openings at the interfaces. Such assumption is in agreement with the observed reduction of the influence of the transport barriers on the overall diffusion process due to temperature increase. In the opposite case of temperature independent transport barriers a few channel or pore openings are expected to be free of any defects, while the remaining openings are completely blocked for diffusing molecules [24,25].

The data in Table 4 show that the value of the intraparticle diffusivity of isooctane in the FAU zeolite is approximately a factor of two higher than that in the EMT zeolite. This observation correlates with the fact that the size of the channels connecting cages is somewhat smaller in the latter zeolite than in the former [10]. For relatively bulky molecules, including isooctane, such difference in the channel sizes can be a reason of a slower diffusion in the EMT zeolite in comparison to that in the FAU zeolite.

## 4. Samples

### 4.1. CMS Membranes

The carbon molecular sieve membranes used in this study were synthesized as described in references [1,5,20]. To briefly summarize, the CMS membranes were formed by synthesis of precursor polymer films and subsequent pyrolysis of the films under carefully controlled conditions. The precursor copolymers used were 6FDA/BPDA-DAM, synthesized in house, with a ratio of 6FDA to BPDA of 1:1 and commercially available Matrimid^®^ (Huntsman). Both are high molecular weight polyimides and have relatively high glass transition temperatures of over 500 K [5,26]. All membranes were pyrolyzed at 823 K in the following prescribed pyrolysis atmospheres: inert Ar atmosphere with <1 ppm of O_2_ for 6FDA/BPDA and Matrimid Sample 1, and under vacuum for Matrimid Sample 2. It was recently demonstrated that the presence of oxygen in the pyrolysis atmosphere can lead to more selective and less permeable ultra-micropores to form in the resulting CMS membranes [1,5,20,26].

### 4.2. Zeolite Samples

The EMT-type zeolite was synthesized as described in [27] using 18-Crown-6 as template. The FAU-type zeolite was formed using a procedure similar to that reported in [28]. The starting materials in the synthesis of the latter zeolite were sodium silicate, sodium aluminate, sodium hydroxide, and water. Synthesis of the FAU/EMT intergrowth was performed using mixed templates of 18-crown-6 and 15-crown-5. The Si-to-Al-ratio calculated from the inductively coupled plasma mass spectrometry (ICP) data ranged from 3 to 6. Powder XRD was used to confirm the formation of the zeolites.

### 4.3. Preparation of Samples for PFG NMR Studies

PFG NMR samples were prepared as follows. Around 200 mg of zeolite, or between 20 and 40 mg of membrane pieces were introduced into 5 mm NMR tubes. The NMR tubes were connected to a custom-made vacuum system and the samples were activated (*i.e.*, made sorbate-free) by applying a high vacuum at an elevated temperature for at least 24 hours. Sorbate loading was performed after sample activation by exposing the microporous samples to the desired sorbate pressure for at least 4 hours at 298 K. Upon loading, the NMR tube was flame sealed and separated from the vacuum system.

For all CMS membrane samples, high purity methane (99+%, Sigma-Aldrich) was loaded at a pressure of 0.5 bar. To increase the signal to noise ratio in the ^13^C PFG NMR measurements, methane with 99% carbon-13 isotopic enrichment was used.

For all zeolite samples the loading pressure of isooctane was equal to 50 mbar. This resulted in the following sorbate concentrations at 298 K: 182 mg/g for the FAU/EMT intergrowth, 229 mg/g for the FAU zeolite, and 207 mg/g for the EMT zeolite. These loadings were estimated by using the isooctane adsorption isotherms (not shown) measured for the three zeolites at 298 K.

## 5. Conclusions

This review demonstrates the high potential of multinuclear PFG NMR at high field and high gradients for obtaining detailed understanding of sorbate transport in microporous materials relevant for applications in separations and catalysis. In particular, comparison of self-diffusion coefficients and activation energies of diffusion observed for the same sorbate in the three carbon molecular sieve membrane samples allowed a deeper understanding of the differences in the pore structure of these samples. The reported PFG NMR data also shows a high degree of uniformity of the membrane transport properties and the related structural properties in a broad range of length scales between a fraction of a micrometer and more than 10 microns. Diffusion studies of FAU/EMT intergrowth and the comparison of the results of these studies with the corresponding transport properties of the pure FAU- and EMT-type zeolites revealed the existence of strong transport barriers inside particles of the intergrowth. These barriers can be formed as a result of a partial blockage of the channel openings at the interfaces between the intergrowth components of the FAU/EMT particles.
